# Comparison of *albicans *vs. non-*albicans *candidemia in French intensive care units

**DOI:** 10.1186/cc9033

**Published:** 2010-05-27

**Authors:** Olivier Leroy, Jean-Paul Mira, Philippe Montravers, Jean-Pierre Gangneux, Olivier Lortholary

**Affiliations:** 1 Service de Réanimation Médicale et des Maladies Infectieuses, Centre Hospitalier Gustave Dron, 135 rue du Président Coty, 59208 Tourcoing, France; 2 Service de Réanimation Médicale, Hôpital Cochin, 27 rue du Faubourg Saint Jacques, Assistance publique, Hôpitaux de Paris, 75014 Paris, France; 3 Université Paris Descartes, INSERM U567, 12 rue de l'école de Médecine, 75006 Paris, France; 4 Département d'Anesthésie-Réanimation Chirurgicale, Centre Hospitalier Universitaire Bichat-Claude Bernard, 46 rue Henri Huchard, Assistance publique, Hôpitaux de Paris, 75018 Paris, France; 5 Université Paris VII, 16 rue Henri Huchard, 75018 Paris, France; 6 Laboratoire de Parasitologie-Mycologie, Centre Hospitalier Universitaire Pontchallou, 2 rue Henri Le Guilloux 35000 Rennes, France; 7 EA SeRAIC 4427, IRSET-Institut de recherche en santé, Environnement et Travail, Université Rennes 1, 2 avenue du Professeur Léon Bernard, 35043 Rennes, France; 8 Université Paris Descartes, 12 rue de l'école de Médecine, 75006 Paris, France; 9 Centre d'infectiologie Necker-Pasteur, CHU Necker-Enfants-Malades, 149 rue Sèvres, Assistance publique, Hôpitaux de Paris, 75015 Paris, France; 10 Institut Pasteur, 211 rue Vaugirard, 75015 Paris, Centre National de Référence de Mycologie et Antifongiques, CNRS URA3012, France

## Abstract

**Introduction:**

Candidemia raises numerous therapeutic issues for intensive care physicians. Epidemiological data that could guide the choice of initial therapy are still required. This analysis sought to compare the characteristics of intensive care unit (ICU) patients with candidemia due to non-*albicans Candida *species with those of ICU patients with candidemia due to *Candida albicans*.

**Methods:**

A prospective, observational, multicenter, French study was conducted from October 2005 to May 2006. Patients exhibiting candidemia developed during ICU stay and exclusively due either to one or more non-*albicans Candida *species or to *C. albicans *were selected. The data collected included patient characteristics on ICU admission and at the onset of candidemia.

**Results:**

Among the 136 patients analyzed, 78 (57.4%) had candidemia caused by *C. albicans*. These patients had earlier onset of infection (11.1 ± 14.2 days after ICU admission vs. 17.4 ± 17.7, *p *= 0.02), higher severity scores on ICU admission (SOFA: 10.4 ± 4.7 vs. 8.6 ± 4.6, *p *= 0.03; SAPS II: 57.4 ± 22.8 vs. 48.7 ± 15.5, *P *= 0.015), and were less often neutropenic (2.6% vs. 12%, *p *= 0.04) than patients with candidemia due to non-*albicans Candida *species.

**Conclusions:**

Although patients infected with *Candida albicans *differed from patients infected with non-*albicans Candida *species for a few characteristics, no clinical factor appeared pertinent enough to guide the choice of empirical antifungal therapy in ICU.

## Introduction

The importance of fungal infections in Intensive Care Units (ICUs) was recently underlined by the EPIC Study II, since fungal agents represented 19% of positive isolates [1]. Moreover, candidemia still raises numerous therapeutic issues to Intensive Care physicians. The relationship between prognosis and early initiation of the adequate antifungal therapy is well established [2-4]. Ideally, adequate therapy must be started much before candidemia is ascertained, therefore much before the causative *Candida *species is identified and its susceptibility to antifungals is known. Broad spectrum antifungals have enriched the therapeutic arsenal in the past few years. The above therapeutic constraints might tempt clinicians to use these agents widely. However, apart from financial aspects, an excessive usage could become deleterious by resulting in the selection of strains with reduced susceptibility.

In a prospective multicenter observational study named AmarCand performed to assess the current epidemiology, management and prognosis of invasive *Candida *infections in French ICUs, we demonstrated that 95.6% of *C. albicans *strains were susceptible to fluconazole whereas only 68% of non-*albicans Candida *were susceptible [5]. Thus, among data that could guide the choice of initial therapy, the availability of elements that would allow a binary distinction, with sufficient liability, between *albicans *or non-*albicans Candida *could represent an interesting first step.

In the present paper, the characteristics of patients from the AmarCand study, on ICU admission and at the onset of candidemia, are described according to whether candidemia was due to an *albicans *or a non-*albicans Candida *strain.

## Materials and methods

### The AmarCand ("Analyse du Management en Anesthésie et Réanimation des Candidoses invasives") study

This study has already been described in two publications from the Group [5,6]. Briefly, AmarCand was a prospective, multicentre, national and observational study. Adult ICU patients with invasive *Candida *infection requiring a systemic antifungal therapy were included. Criteria used for diagnosis were those proposed in 2002 by the members of the European Organization for Research and Treatment of Cancer/Invasive Fungal Infections Cooperative Group and the National Institute of Allergy and Infectious Diseases Mycoses Study Group [7]. In accordance with the French law, approval of an Ethics Committee was not required. However, all patients gave informed consent to participate. Approval of the "Commission Nationale de l'Informatique et des Libertés" was obtained, ensuring that patient data were kept confidential according to the French regulation.

For each episode of invasive *Candida *infection, demographic characteristics, underlying diseases, current hospitalization, severity of illness, and process of care were recorded by each investigator on a standardized report form. Identification of the *Candida *isolates was performed in mycology and microbiology laboratories using the routine methods of each hospital. Isolates were classified as susceptible (S), susceptible-dose dependent (S-DD), or resistant (R) to antifungals according to CLSI interpretive categories [8].

### Comparison of candidemia due to *Candida albicans *vs. non-*albicans Candida *species

For this comparison, we identified patients from the AmarCand study with a candidemia acquired in ICU and exclusively due either to *C. albicans *or to one or more non-*albicans Candida *species. These groups were compared for the patients' characteristics on ICU admission and at the onset of candidemia.

### Statistics

Data were analyzed using SAS^® ^8.2 (SAS Institute Inc., Cary, NC, USA). Variables were expressed as mean values ± standard deviation for numerical variables and as frequencies and percentages for categorical variables. Groups were compared using the Chi-square and Fisher's exact tests. Continuous variables were compared using the Student's *t *test. Statistical significance was accepted at the 5% level.

## Results

A total of 271 evaluable patients were included in the AmarCand study between October 2005 and May 2006. A total of 101 ICUs participated: 44 (43.6%) medico-surgical ICUs, 28 (27.7%) medical ICUs and 29 (28.7%) surgical ICUs.

For the purposes of the present paper, we excluded from the 271 evaluable patients: 87 patients with invasive candidiasis but no candidemia, 13 patients with mixed candidemia due to *albicans *and non-*albicans Candida *species, and 35 patients who acquired candidemia before admission in ICU. Therefore, 136 patients were included in the present analysis.

Candidemia was due to *C. albicans *in 78 (57.4%) patients. It was due to non-*albicans Candida *species in 58 (42.6%) patients. In total, 63 non-*albicans Candida *isolates were identified: *C. glabrata *n = 25, *C. parapsilosis *n = 12, *C. tropicalis *n = 9, *C. kefyr *n = 4, *C. krusei *n = 6 and other species n = 7. *In vitro *susceptibility to fluconazole was determined for 112 isolates. The rate of fluconazole-R or S-DD *Candida *was 3.3% (2/61) for *C. albicans*, 50.0% (10/20) for *C. glabrata*, 18.2% (2/11) for *C. parapsilosis*, 100% (3/3) for *C. krusei*, 25% (2/8) for *C. tropicalis*, 0% (0/4) for *C. kefyr*, and 40% (2/5) for the remaining *Candida *species. Susceptibility to fluconazole was determined for 61 episodes of candidemia due to *C. albicans *and for 47 episodes due to non-*albicans Candida *species. The rate of episodes due to a fluconazole-R or S-DD *Candida *was 3.3% and 38.3%, respectively (*P *< 0.0001).

Table 1 provides the major characteristics of patients on admission in ICU. The only significant differences observed between both types of candidemia were the Simplified Acute Physiology Score II (SAPS II) and the Sepsis-related Organ Failure Assessment (SOFA) score, which were significantly higher in case of infection with *C. albicans*.

**Table 1:** Clinical characteristics of patients on admission to intensive care unit

	Total N = 136	Candidemia due to *C. albicans* N = 78	Candidemia due to non-*albicans Candida *species N = 58	*P *value
Age (years)	62.1 ± 14.9	61.0 ± 17.2	63.5 ± 11.1	0.32
Male gender	84 (61.8)	45 (57.7)	39 (67.2)	0.26
SAPS II	53.8 ± 20.4	57.4 ± 22.8	48.7 ± 15.5	0.015
SOFA	9.6 ± 4.7	10.4 ± 4.7	8.6 ± 4.6	0.03
Underlying disease*				0.35
Absent or nonfatal	63 (46.3)	37 (47.4)	26 (44.8)	
Ultimately fatal	58 (42.7)	35 (44.9)	23 (39.7)	
Rapidly fatal	15 (11.0)	6 (7.7)	9 (15.5)	
Chronic renal failure	26 (19.1)	16 (20.5)	10 (17.2)	0.63
Type 1 diabetes mellitus	16 (11.8)	8 (10.3)	8 (13.8)	0.52
Solid neoplastic tumor	32 (23.5)	19 (24.4)	13 (22.4)	0.79
Hematological malignancy	7 (5.1)	4 (5.1)	3 (5.2)	0.99
Immunosuppression	28 (20.6)	15 (19.2)	13 (22.4)	0.65
Corticotherapy	10 (7.4)	3 (3.8)	7 (12.1)	0.06
HIV infection	2 (1.5)	2 (2.6)	0	0.17
Cancer chemotherapy	10 (7.4)	5 (6.4)	5 (8.6)	0.78
Organ transplantation	5 (3.7)	2 (2.6)	3 (5.2)	0.50
Immunosuppressant therapy	4 (2.9)	2 (2.6)	2 (3.4)	0.88
Intravenous drug use	2 (1.5)	1 (1.3)	1 (1.7)	0.83
Neutropenia (<500/mm^3^)	9 (6.6)	2 (2.6)	7 (12.1)	0.04

Results are expressed as mean ± SD values or numbers (%) of patients.

HIV: human immunodeficiency virus; SAPS: simplified acute physiology score; SOFA: sepsis-related organ failure assessment

*Classified according to the criteria proposed by McCabe and Jackson [17]

The time from ICU admission to onset of candidemia was 13.8 ± 16.1 days. It was significantly shorter in the case of candidemia due to *C. albicans*: 11.1 ± 14.2 days vs. 17.4 ± 17.7 days with non-*albicans Candida *species (*P = *0.02). Candidemia developed within six days after admission in ICU for 59 patients. Such an early infection was significantly more frequent when candidemia was due to *C. albicans *than when it was due to non-*albicans Candida *species (40/78 vs. 19/58, *P = *0.03). Neutropenia (absolute neutrophil count <500 cells/mm^3^) was concomitant to candidemia in nine patients. It was significantly more frequent when candidemia was caused by non-*albicans Candida *species than when it was caused by *C. albicans *(7/58 vs. 2/78, *P = *0.04).

The main features of the patients' care at the onset of candidemia are shown in Table 2. There were no significant differences between the two patient groups, notably for previous exposure to azole agents.

**Table 2:** Main features of patients' care at the onset of candidemia

	Total N = 136	Candidemia due to *C. albicans* N = 78	Candidemia due to non-*albicans Candida *species N = 58	*P *value
Recent surgery (<3 months)	75 (55.1)	43 (55.1)	32 (55.2)	0.99
Intra-abdominal	56 (41.2)	32 (41.0)	24 (41.6)	0.95
Vascular	9 (6.6)	6 (7.7)	3 (5.2)	0.54
Time from surgery to candidemia (days)	23.6 ± 20.7	22.6 ± 19.3	25.0 ± 22.7	0.62
Invasive mechanical ventilation	106 (77.9)	62 (79.5)	44 (75.9)	0.61
CVC	122 (89.8)	68 (87.2)	54 (93.2)	0.26
Time from CVC placement to candidemia (days)	15.2 ± 16.9	13.1 ± 14.9	17.8 ± 18.9	0.13
UC	122 (89.8)	68 (87.2)	54 (63.2)	0.26
Time from UC placement to candidemia (days)	16.4 ± 17.0	14.7 ± 16.7	18.6 ± 17.4	0.22
Prior antibiotherapy	84 (61.8)	48 (61.5)	36 (62.1)	0.95
Duration of antibiotherapy before candidemia (days)	18.2 ± 13.5	17.8 ± 14.4	18.7 ± 12.4	0.77
Previous exposure to azole agents	23 (16.9)	11 (14.1)	12 (20.7)	0.31
Vasoactive drug use	31 (22.8)	20 (25.6)	11 (19.0)	0.36

Results are expressed as mean ± SD values or numbers (%) of patients. CVC: central venous catheter; UC: urinary catheter

## Discussion

In this study, the characteristics of patients with candidemia caused by non-*albicans Candida *species versus *Candida albicans *in ICU were compared. The main result is that only a few significant differences were observed: severity of the disease, the time to candidemia onset and the rate of underlying neutropenia. So, we did not identify a parameter pertinent enough to allow a binary distinction between *albicans *or non-*albicans Candida *and to guide the choice of empirical antifungal therapy.

Studies that compare the epidemiological characteristics of patients with candidemia caused by non-*albicans Candida *species versus *Candida albicans *are scarce and their results are disparate.

Three studies included patients who were not all adults and/or not all admitted in ICU. Cheng *et al*. retrospectively analyzed 130 cases of fatal candidemia due to either a *C. albicans *(n = 68) or a non-*albicans C*. species (n = 62) [9]. Multivariate analyses showed that factors independently associated with *C. albicans *infection were the age ≥65 years, hyperleukocytosis (>15,000 cells/mm^3^), and immunosuppressant therapy. Shorr *et al*. retrospectively analyzed the files of 245 candidemic patients from two different hospitals [10]. *C. albicans *represented 52% of the causative species. None of the parameters describing severity of the disease and previous exposition to azole agents was significantly predictive of a candidemia due to a non-*albicans Candida *strain. The third report arises from a large registry of 2,019 patients included between July 2004 and March 2008 in 23 North American hospitals [11]. Underlying hematological malignancy and bone marrow grafting were less common in patients with a candidemia due to *C. albicans*. Prior antifungal therapy was reported in 43% of the 2,019 patients. It was significantly less frequent in patients with *C. albicans *infection (38.8% vs. 46.5%, *P *< 0.001).

Three other studies were performed exclusively in ICU. In Australia Playford *et al*. carried out a three-year prospective, national survey [12]. A total of 179 episodes of candidemia were studied, of which 62% were related to *C. albicans*. Factors associated independently with a candidemia not related to *C. albicans *were recent intra-abdominal surgery and recent exposition to systemic antifungal therapy. Chow *et al*. compared 79 patients with candidemia due to *C. albicans *and 67 patients with candidemia due to non-*albicans Candida *species [13]. Previous exposition to azole agents, duration of central venous catheter implantation and the number of antimicrobial agents per day were associated with non-*albicans Candida *infection in multivariate analyses. Conversely, the duration of parenteral nutrition was associated with a reduced risk of non-*albicans Candida *infection. Finally, 189 candidemic patients (*C. albicans*: 56%) were included in the international, multicenter, retrospective study of Holley *et al. *[14]. Factors associated independently with candidemia due to non-*albicans Candida *species were female gender and duration of central venous catheter implantation using multivariate analysis.

Our results obtained in 136 episodes of candidemia contrast with those of the three above studies performed in ICU [12-14]. Indeed, neither intra-abdominal surgery, previous exposure to azole agents, duration of central venous catheter implantation, nor female gender were associated with non-*albicans Candida *infection. Our results are however in line with those of Shorr *et al*., who could not identify clearly any parameter associated with the *Candida *species causative of candidemia [10].

Several consensual recommendations for the management of invasive *Candida *infections have been published in the last few years. In the French recommendations, which go back to 2004, the algorithm takes into account previous exposition to azole agents [15]. Empirical treatment based on fluconazole is proposed for patients who had not been exposed previously to azole agents. The 2009 North American recommendations propose empirical broad-spectrum treatment with an echinocandin for all ICU patients irrespective of previous exposition to azole agents [16]. Both recommendations propose to continue therapy with de-escalation if the causative strain is susceptible to fluconazole. Our results showing a high incidence (38.3%) of fluconazole non-susceptible *Candida*, and no pertinent parameter able to predict the species of causative *Candida *suggest that, in France and probably the rest of Europe, the use of the North American recommendation may now be more adequate. The use of echinocandin as first-line treatment (before the identification of the causative *Candida *strain and determination of its susceptibility) for all ICU patients suffering from candidemia could decrease the incidence of inappropriate empirical antifungal therapy and thus improve outcome of patients with such invasive candidiasis [3]. A de-escalation could be proposed in patients who are clinically stable when minimum inhibitory concentration (MIC) to fluconazole is ≤8 mg/L [16].

## Conclusions

Comparison of the characteristics of ICU patients with candidemia caused by non-*albicans Candida *species versus *Candida albicans *did not allow identifying any parameter pertinent enough to guide the choice of empirical antifungal therapy.

## Key messages

• Characteristics of patients with candidemia caused by non-*albicans Candida *species versus *Candida albicans *are quite similar at the onset of candidemia.

• Empiric antifungal therapy should be based on a broad-spectrum treatment effective against non-*albicans Candida *species and *Candida albicans*.

## Abbreviations

AmarCand: "Analyse du Management en Anesthésie et Réanimation des Candidoses invasives"; *C: Candida; *CVC: central venous catheter; HIV: human immunodeficiency virus; ICU: intensive care unit; MIC: minimum inhibitory concentration; SAPS: simplified acute physiology score; SOFA: sepsis-related organ failure assessment; UC: urinary catheter.

## Competing interests

OLer has received speaking honoraria from Pfizer, MSD, Astellas, Schering Plough; JPM has received speaking honoraria from Pfizer, MSD, Astellas; PM has received speaking honoraria from Pfizer, MSD, Astellas, Astra Zeneca, Eli Lilly; JPG has received speaking honoraria from Pfizer, MSD, Astellas, Schering Plough, Gilead Sciences; and OLor has received speaking honoraria from Pfizer, MSD, Astellas, Schering Plough, and Gilead Sciences.

The research, including the article-processing charge, was funded in full by Merck Sharpe & Dohme-Chibret, France.

## Authors' contributions

OLer contributed to the design of the study and wrote the manuscript. JPM contributed to the design of the study and contributed to the final revision of the manuscript for important intellectual content. PM contributed to the design of the study and contributed to the final revision of the manuscript for important intellectual content. JPG contributed to the design of the study and contributed to the final revision of the manuscript for important intellectual content. OLor contributed to the design of the study and contributed to the final revision of the manuscript for important intellectual content. All the authors read and approved the final manuscript.
